# Postural control learning dynamics in Parkinson’s disease: early improvement with plateau in stability, and continuous progression in flexibility and mobility

**DOI:** 10.1186/s12938-020-00776-1

**Published:** 2020-05-11

**Authors:** Zahra Rahmati, Saeed Behzadipour, Alfred C. Schouten, Ghorban Taghizadeh, Keikhosrow Firoozbakhsh

**Affiliations:** 1 Mechanical Engineering Department, Sharif University of Technology, Tehran, Iran; 2 Djawad Movafaghian Research Center in Neurorehab Technologies, Sharif University of Technology, Tehran, Iran; 3 Department of Biomechanical Engineering, Delft University of Technology, Delft, The Netherlands; 4 Department of Biomechanical Engineering, University of Twente, Enschede, The Netherlands; 5 Rehabilitation Research Center, Department of Occupational Therapy, School of Rehabilitation Sciences, Iran University of Medical Sciences, Tehran, Iran

**Keywords:** Postural control model, Parkinson’s disease, Learning dynamics, Pattern of improvement, Stability and flexibility degree

## Abstract

**Background:**

Balance training improves postural control in Parkinson’s disease (PD). However, a systematic approach for the development of individualized, optimal training programs is still lacking, as the learning dynamics of the postural control in PD, over a training program, are poorly understood.

**Objectives:**

We investigated the learning dynamics of the postural control in PD, during a balance-training program, in terms of the clinical, posturographic, and novel model-based measures.

**Methods:**

Twenty patients with PD participated in a balance-training program, 3 days a week, for 6 weeks. Clinical tests assessed functional balance and mobility pre-training, mid-training, and post-training. Center-of-pressure (COP) was recorded at four time-points during the training (pre-, week 2, week 4, and post-training). COP was used to calculate the sway measures and to identify the parameters of a patient-specific postural control model, at each time-point. The posturographic and model-based measures constituted the two sets of stability- and flexibility-related measures.

**Results:**

Mobility- and flexibility-related measures showed a continuous improvement during the balance-training program. In particular, mobility improved at mid-training and continued to improve to the end of the training, whereas flexibility-related measures reached significance only at the end. The progression in the balance- and stability-related measures was characterized by early improvements over the first 3 to 4 weeks of training, and reached a plateau for the rest of the training.

**Conclusions:**

The progression in balance and postural stability is achieved earlier and susceptible to plateau out, while mobility and flexibility continue to improve during the balance training.

## Background

Parkinson’s disease (PD) is a progressive neurodegenerative disorder, which is traditionally managed by symptomatic treatments [1]. Among motor and non-motor manifestation of PD, axial (gait and posture) symptoms evolve more rapidly [2]. As PD progresses, non-dopaminergic motor circuits are also involved, exacerbating the axial motor features that do not usually respond to standard antiparkinsonian medication [3, 4]. Gradual deterioration of muscle strength, balance, and gait causes postural instability and immobility [5], which considerably diminish quality of life, and are known as risk factors for fall [6, 7]. Several studies suggested rehabilitation as an adjuvant to pharmacological and surgical treatments [1, 8], which is proven to slow down the progression of PD and act as a neuroprotective strategy [9–11].

Although it is well evidenced that the physical exercises counteract the motor degradation (especially balance and gait) in patients with PD [12, 13], still many open questions remain regarding the optimal intervention. Training programs are prescribed based on empirical experiences [1] and a definite rationale for development of individualized and impairment-based interventions is still lacking [14, 15]. Several studies compared different training programs (e.g., resistance, balance, treadmill training) [4, 13], or investigated the effects of specific training modality on various clinical outcomes [16, 17]. In addition, numerous reviews and meta-analysis were carried out on randomized controlled clinical trials (RCT) to recommend evidence-based exercise guidelines [1, 12, 18–22]. However, theses reviews all indicate that there is a broad heterogeneity in RCTs regarding the optimal delivery (dosage, frequency, duration), and content of exercises (specificity, complexity, needed modalities) for each targeted stage of the disease. Apart from heterogeneity among RCTs, most RCTs used multicomponent training programs as well as insensitive and multidimensional assessments, which further caused these reviews to be inconclusive [4, 8, 23]. These studies highlight the need for disclosing the dose–response relationship for improvement of postural control as a result of different training modalities and exercise components [8, 22]. Furthermore, the most sensitive and well-defined clinical measures to assess the effect of trainings on postural control are still undetermined [2, 4].

The further we gain knowledge about the learning dynamics of postural control during a training program, and in particular, balance training, the closer we come to an answer for an optimal patient-specific training regimen. Design of an optimal balance-training program needs adjusting optimal number of training sessions (neither lengthy, exhausting and in vain, nor insufficient and ineffective), targeted exercise components, and sufficient intensity for each exercise component. An essential and first step toward such approach is to gain prior knowledge about all these factors, or particularly, to understand the learning dynamics of postural control during different training programs. Nonetheless, the dynamics of the postural control motor learning is relatively unknown due to the paucity of longitudinal studies with multipoint assessments, over a course of training. The majority of RCTs are designed with assessments at baseline and follow-up after intervention; and only a few used intermediate assessments during a training program [24–26]. Multipoint-assessment design is generally used to evaluate the follow-up lasting effects of an applied surgical [3] or physical [6, 27] therapy, or to investigate the natural progression of L-dopa-treated PD [2, 7]. To the best of our knowledge, there is no study which investigated the learning dynamics in postural control during a balance-training program. Some studies suggest that the learning rates in dual-tasking or in upper extremities functions in PD patients are reduced compared to healthy subjects [1, 13, 28]. Peterson et al. [28] also found that people with PD have different learning dynamics and retention pattern when exposed to translational perturbation in 1 day and re-exposed the next day for assessment. Yet, these patients were not involved in a training program. Therefore, a longitudinal study of postural control learning dynamics based on the sensitive and quantitative measures is highly demanded.

Moreover, to deliver a patient-specific balance-training program, a framework with unidimensional measures is needed to quantitatively define each patient’s initial and ongoing state of the postural control performance, which is still lacking in the literature. Furthermore, given the many contributing factors to postural control (e.g., flexibility, strength, balance) as well as the inefficiency of clinimetric measurements provided yet, inconsistent results may arise in the investigations of postural control learning dynamics. For instance, flexibility, as opposed to ‘rigidity’ [29], denotes the involvement of higher degrees of freedom in postural control [30]. As such, flexibility and stability concurrently contribute in postural control, which made some researchers to investigate the contribution of each one, and particularly, the extent of this contribution in postural control (in response to surface perturbations) [31–33] as well as functional disabilities [34, 35] in PD. Yet, this contribution was not unveiled with a quantitative and unidimensional measure. In our previous study, we proposed a computational framework, which disentangles the ‘stability’ and ‘flexibility’ degree—denoted by *K*_*P*_ and *K*_*n*_, respectively—in patients with PD. The framework was based on general postural sway measures, which in turn were earlier shown to be sensitive to different types of training programs [23]. Moreover, the framework showed to be sufficiently sensitive to balance-training programs [36, 37], and as such paved the path for the future studies of postural control learning dynamics, using unidimensional and meaningful assessment measures.

In this study, we investigated the learning dynamics of postural control in PD during a balance-training program, and as such introduced a systematic approach for future design of optimal balance-training programs. In particular, we used the unidimensional measures that we previously proposed [37] based on a patient-specific postural control model of PD. For this purpose, a representative PD cohort receiving a 6-week balance-training program was assessed clinically and experimentally at multiple time points during the training. Finally, the patterns for all experimental measures were addressed in conjunction with the correspondent patterns in clinical measures; thereby providing recommendations for future prospect of optimal exercise guidelines for PD.

## Results

The results of the multipoint clinical and experimental assessments of the patients with PD, who participated in the 6-week (18-session) balance-training program, are presented in this section.

### Clinical outcomes

The results of the clinical assessments at pre-, mid-, and post-training are provided in Table 1, including the statistical results. Patients were assessed at three time points during balance training (pre-, mid-, and post-training). The clinical tests assessed the functional balance and mobility of patients, as shown in Table 1.

**Table 1:** Clinical outcomes of patients with PD at pre-, mid-, and post-training

Clinical measure	PD patients (n = 20)			ANOVA *P* value (*F* value)	Effect size	Tukey *P* value for post-hoc comparisons			Change pattern
	Pre-training	Mid-training	Post-training			Pre to mid	Mid to post	Pre to post	
Functional balance									
Functional reach test (cm)	23.5 ± 7.9	32.8 ± 6.7*	37.6 ± 6.1^†^*	*< 0.0001* (43.1)	0.694	*<0.0001*	*<0.0005*	*<0.0001*	Continuous
Step test (taps in 15 s)	13.2 ± 3.5	15.9 ± 4.0*	17.3 ± 3.6*	*< 0.0001* (23.9)	0.557	*<0.0001*	0.063	*<0.0001*	Saturation
Tinetti balance score	14.7 ± 1.5	15.6 ± 0.9*	15.9 ± 0.2*	*< 0.0005* (9.44)	0.332	*0.033*	0.320	*0.011*	Saturation
Tandem stance^a^—EO (s)	93.0 ± 27.6	113.7 ± 12.6*	118.3 ± 5.7*	*< 0.0001* (14.5)	0.433	*0.003*	0.141	*0.002*	Saturation
Tandem stance^a^—EC (s)	35.4 ± 26.8	54.8 ± 29.1*	72.7 ± 30.2^†^*	*< 0.0001* (23.2)	0.549	*0.004*	*0.012*	*< 0.0001*	Continuous
Functional mobility									
TUG (s)	9.1 ± 2.7	7.4 ± 1.6*	6.5 ± 1.4*^†^	*< 0.0001* (23.5)	0.553	*0.0007*	*0.004*	*0.0001*	Continuous
6MWT (m)	226.0 ± 67	254.1 ± 61*	305.5 ± 62*^†^	*< 0.0001* (19.8)	0.510	*0.040*	*0.002*	*0.0001*	Continuous
Tinetti gait score	10.5 ± 1.4	11.5 ± 0.6*	11.8 ± 0.4*^†^	*< 0.0001* (13.8)	0.422	*0.009*	*0.005*	*0.001*	Continuous

Values are reported as mean ± standard deviation

EO, eyes open; EC, eyes closed; TUG, Timed Up and Go test; 6MWT, Six-minute walk test; FRT, Functional reach test; Continuous, continuously improving with significant difference between all time points; Saturation, improvements with saturation at the end—i.e., significant change in the first half of the training (from pre- to mid-training), but then non-significant from mid- to post-training points

Post-hoc Tukey tests for pairwise comparisons between time points: *significantly different from pre-training (*P* < 0.05); ^†^significantly different from mid-training (*P* < 0.05)

Significant *P* values are in italic

^a^Timed tandem stance was performed with the right and left leg in the front position, and then the time of both legs was summed as one scale (with maximum score of 120 s, considering that the maximum time to complete each stance test was set to 60 s)

All measures of functional balance and mobility improved after balance training. The improvement pattern was either continuous with significant difference between all time points (Continuous) or the improvement was observed only at the first part of the training (significant from pre- to mid-training), and came to a saturation for the rest, i.e., non-significant from mid- to post-training (Saturation). All the mobility tests (TUG, 6MWT, Tinetti gait score) exhibited a continuous improvement. In contrast, most of balance tests (i.e., Step test, Tinetti balance score, Tandem stance—EO) presented the Saturation pattern. A few balance tests (i.e. FRT and Tandem stance—EC), however, presented the Continuous pattern.

### Experimental and model-based outcomes

In addition to clinical assessments, the center-of-pressure (COP) was recorded at four time points during the balance training (i.e., pre-, week 2, week 4, and post-training); the results of which are presented in Tables 2, and 3. Table 2 shows the results for two tasks on rigid surface (R-task: RO, RC); and Table 3 shows the results for tasks on foam (F-tasks: FO, FC). The results include the four sway measures, which were extracted from the COP (i.e., root mean square, *RMS*, mean velocity, *MV*; the frequency up to which 95% of the total power lies, *f95*; and the time coordinate of the critical point in stabilogram diffusion function diagram, ∆*t*_*c*_). In addition, the parameters of a patient-specific postural control model in the form of an inverted pendulum, a PID controller (*K*_*P*_, proportional gain, or stability degree; *K*_*D*_, damping of the ankle joint; *K*_*I*_, the integral gain) with time delay (*τ*_*d*_), as well as the sway scaling gain (*K*_*N*_—flexibility degree) were calculated and are reported in these tables. In particular, the flexibility-related measures (*MV*, *K*_*N*_) showed changes after training in R-tasks, and the stability-related measures (*f95*, ∆*t*_*c*-_, *K*_*P*_) changed in F-tasks, as stated in the following.

**Table 2:** Sway measures (*RMS*, *MV*, *f95*, ∆*t*_*c*_) and model parameters (*K*_*P*_, *K*_*D*_, *K*_*I*_, *K*_*n*_, *τ*_*d*_) of patients with PD, at pre-, week 2, week 4, and post-training, in R-tasks (RO: stance on rigid surface with eyes open, and RC: stance on rigid surface with eyes closed)

Task	PD patients (*n* = 20)				ANOVA *P* value (*F* value)	Effect size	Tukey *P* value for post-hoc comparisons					
Sway measures/model parameters	Pre (*T*1)	Week 2 (T2)	Week 4 (T3)	Post (T4)			*T*1–*T*2	*T*1–*T*3	*T*1–*T*4	*T*2–*T*3	*T*2–*T*4	*T*3–*T*4
RO												
*RMS* (mm)	5.99 ± 1.80	7.21 ± 2.90	6.78 ± 2.38	6.56 ± 1.98	0.186 (1.66)	0.080	0.346	0.562	0.381	0.842	0.716	0.970
*MV* (mm/s)	10.04 ± 3.25	10.22 ± s3.75	11.20 ± 3.41	12.31 ± 4.30^†^	*0.010* (4.13)	0.179	0.994	0.468	0.052	0.266	*0.019*	0.541
*f95* (Hz)	1.14 ± 0.39	1.12 ± 0.35	1.26 ± 0.42	1.37 ± 0.58	0.106 (2.13)	0.101	0.998	0.712	0.243	0.373	0.260	0.792
∆*t*_*c*_ (s)	1.59 ± 0.54	1.75 ± 0.57	1.70 ± 0.41	1.76 ± 0.49	0.531 (0.742)	0.038	0.686	0.821	0.452	0.983	1.000	0.935
*K*_*P*_ (N.m/deg)	16.43 ± 3.78	16.84 ± 3.65	16.96 ± 3.51	18.42 ± 4.88	0.062 (2.58)	0.120	0.958	0.750	0.192	0.998	0.079	0.383
*K*_*D*_ (N.m.s/deg)	5.87 ± 1.84	5.22 ± 1.93	5.47 ± 1.54	5.94 ± 2.20	0.370 (1.07)	0.053	0.309	0.802	1.000	0.931	0.372	0.788
*K*_*I*_ (N.m/deg/s)	1.46 ± 0.82	1.09 ± 0.74	1.56 ± 0.65	1.31 ± 0.76	0.125 (2.00)	0.095	0.192	0.971	0.916	0.187	0.690	0.436
*K*_*n*_	446.9 ± 215	462.3 ± 214	543.3 ± 211	568.9 ± 197*	*0.022* (3.48)	0.155	0.989	0.278	*0.036*	0.132	0.085	0.956
*τ*_*d*_ (ms)	135.3 ± 33.0	115.7 ± 44.0	117.1 ± 28.6	109.3 ± 28.5	0.059 (2.63)	0.122	0.339	0.294	0.058	0.999	0.914	0.768
RC												
*RMS* (mm)	6.64 ± 2.11	7.13 ± 3.09	7.23 ± 2.42	6.63 ± 2.10	0.463 (0.868)	0.044	0.827	0.428	1.000	0.998	0.850	0.201
*MV* (mm/s)	11.94 ± 5.37	11.73 ± 5.37	13.81 ± 5.08	14.92 ± 6.12*	*0.034* (3.09)	0.140	0.999	0.109	*0.047*	0.360	0.216	0.725
*f95* (Hz)	1.37 ± 0.51	1.47 ± 0.53	1.56 ± 0.52	1.74 ± 0.70	0.085 (2.31)	0.109	0.802	0.472	0.215	0.903	0.389	0.599
∆*t*_*c*_ (s)	1.51 ± 0.59	1.22 ± 0.37	1.23 ± 0.35	1.34 ± 0.47	0.093 (2.24)	0.105	0.180	0.246	0.676	0.998	0.780	0.637
*K*_*P*_ (N.m/deg)	19.64 ± 6.57	18.91 ± 4.62	19.49 ± 5.91	21.13 ± 5.53	0.148 (1.85)	0.089	0.874	0.999	0.354	0.914	0.061	0.474
*K*_*D*_ (N.m.s/deg)	6.06 ± 2.26	5.84 ± 1.28	6.45 ± 1.72	6.69 ± 2.12	0.110 (2.10)	0.100	0.958	0.637	0.463	0.214	0.199	0.865
*K*_*I*_ (N.m/deg/s)	1.83 ± 1.37	1.60 ± 0.87	1.66 ± 0.94	2.05 ± 1.09	0.264 (1.36)	0.067	0.831	0.934	0.866	0.991	0.166	0.199
*K*_*n*_	547.1 ± 314	568.8 ± 303	652.1 ± 321	718.0 ± 344	*0.035* (3.07)	0.139	1.000	0.575	0.120	0.238	0.071	0.486
*τ*_*d*_ (ms)	121.9 ± 40.4	129.8 ± 40.3	127.6 ± 38.1	117.0 ± 41.1	0.428 (0.939)	0.047	0.876	0.907	0.929	0.993	0.456	0.290

Values are reported as mean ± standard deviation. Significant *P* values are in italics

*T*1 to *T*4 refer to pre-, week 2, week 4, and post-training, respectively

* Significantly different from pre-training (*P* < 0.05); ^†^significantly different from week 2 (*P* < 0.05)

**Table 3:** Sway measures (*RMS*, *MV*, *f95*, ∆*t*_*c*_) and model parameters (*K*_*P*_, *K*_*D*_, *K*_*I*_, *K*_*n*_, *τ*_*d*_) of patients with PD, at pre-, week 2, week 4, and post-training, in F-tasks (FO: stance on foam with eyes open, and FC: stance on foam with eyes closed)

Task	PD patients (*n* = 20)				ANOVA *P* value (*F*-value)	Effect size	Tukey *P* value for post-hoc comparisons					
Sway measures/model parameters	Pre (*T*1)	Week 2 (*T*2)	Week 4 (*T*3)	Post (*T*4)			*T*1–*T*2	*T*1–*T*3	*T*1–*T*4	*T*2–*T*3	*T*2–*T*4	*T*3–*T*4
FO												
* RMS* (mm)	10.72 ± 2.89	9.20 ± 2.47	9.73 ± 2.21	9.37 ± 1.98	*0.041* (2.94)	0.134	0.105	0.397	0.227	0.674	0.987	0.840
* MV* (mm/s)	19.80 ± 6.52	19.17 ± 6.30	19.89 ± 5.82	18.51 ± 4.70	0.616 (0.603)	0.031	0.944	1.000	0.631	0.933	0.937	0.629
* f95* (Hz)	1.05 ± 0.24	1.12 ± 0.22	1.31 ± 0.33*^†^	1.32 ± 0.35*	*0.0001* (8.11)	0.299	0.549	*0.016*	*0.020*	*0.026*	0.059	0.996
∆*t*_*c*_ (s)	1.58 ± 0.44	1.43 ± 0.45	1.21 ± 0.25*	1.31 ± 0.28*	*0.003* (5.35)	0.220	0.648	*0.006*	*0.043*	0.116	0.666	0.308
* K*_*P*_ (N.m/deg)	18.52 ± 4.34	18.69 ± 5.21	20.49 ± 5.78	19.75 ± 5.21	*0.021* (3.50)	0.156	0.992	0.123	0.276	0.237	0.397	0.299
* K*_*D*_ (N.m.s/deg)	5.23 ± 1.65	5.12 ± 1.46	5.34 ± 1.17	5.49 ± 1.33	0.602 (0.625)	0.032	0.990	0.990	0.844	0.824	0.088	0.868
* K*_*I*_ (N.m/deg/s)	1.93 ± 1.21	1.96 ± 1.36	1.88 ± 0.82	1.89 ± 1.17	0.986 (0.048)	0.003	1.000	0.992	0.999	0.983	0.995	1.000
* K*_*n*_	803.6 ± 262	787.5 ± 259	804.8 ± 172	817.5 ± 208	0.920 (0.164)	0.009	0.988	1.000	0.983	0.981	0.934	0.980
* τ*_*d*_ (ms)	134.8 ± 34.8	125.1 ± 27.2	123.8 ± 31.7	111.9 ± 23.8	*0.032* (3.15)	0.142	0.554	0.680	0.072	0.998	0.053	0.295
FC												
* RMS* (mm)	14.14 ± 2.83	13.40 ± 3.34	12.46 ± 2.33*	12.40 ± 2.81*	*0.013* (3.90)	0.170	0.636	*0.018*	*0.039*	0.532	0.427	0.999
* MV* (mm/s)	29.15 ± 7.83	26.56 ± 7.57	28.07 ± 8.34	26.40 ± 7.22	0.096 (2.22)	0.105	0.128	0.845	0.171	0.548	0.999	0.670
* f95* (Hz)	1.23 ± 0.34	1.21 ± 0.24	1.42 ± 0.34^†^	1.44 ± 0.43	*0.002* (5.81)	0.234	0.974	0.065	0.106	*0.014*	0.076	0.988
∆*t*_*c*_ (s)	1.43 ± 0.40	1.27 ± 0.26	1.18 ± 0.17	1.11 ± 0.15*	*0.001* (6.59)	0.258	0.221	0.061	*0.016*	0.488	0.091	0.576
* K*_*P*_ (N.m/deg)	18.82 ± 4.43	18.69 ± 5.10	20.34 ± 4.82*	20.12 ± 5.30	*0.046* (2.84)	0.130	0.999	*0.042*	0.105	0.277	0.415	0.971
* K*_*D*_ (N.m.s/deg)	5.17 ± 1.54	5.29 ± 1.93	5.27 ± 1.57	5.80 ± 1.43	0.120 (2.03)	0.097	0.950	0.967	0.051	1.000	0.580	0.256
* K*_*I*_ (N.m/deg/s)	2.14 ± 1.19	2.14 ± 1.08	1.97 ± 1.07	2.51 ± 1.54	0.317 (1.20)	0.060	1.000	0.935	0.614	0.934	0.652	0.248
* K*_*n*_	1273 ± 499	1113 ± 493	1153 ± 415	1145 ± 372	0.298 (1.26)	0.062	0.510	0.290	0.608	0.967	0.969	1.000
* τ*_*d*_ (ms)	122.8 ± 37.8	124.6 ± 62.8	125.1 ± 48.5	125.1 ± 46.5	0.994 (0.026)	0.001	0.998	0.996	0.995	1.000	1.000	1.000

Values are reported as mean ± standard deviation. Significant *P* values are in italics

*T*1 to *T*4 refer to pre-, week 2, week 4, and post-training, respectively

*Significantly different from pre-training (*P* < 0.05); ^†^significantly different from week 2 (*P* < 0.05)

**Fig. 1:**
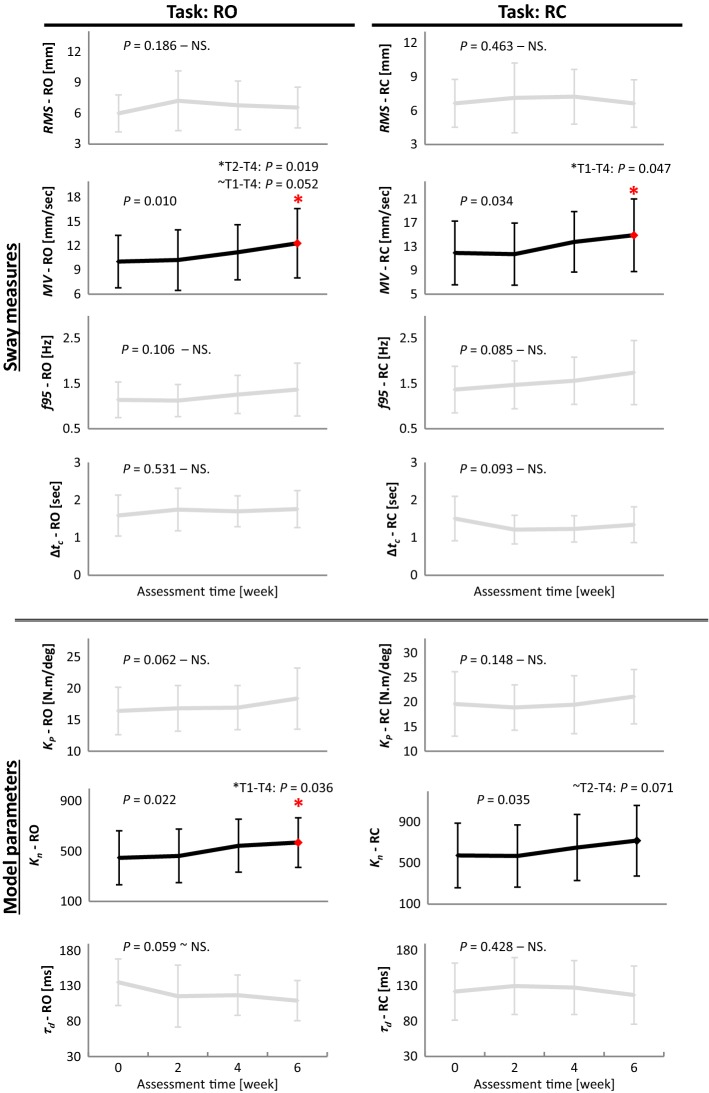
The pattern of improvements for sway measures (*RMS*, *MV*, *f95*, ∆*t*_*c*_) and model parameters (*K*_*P*_, *K*_*n*_, *τ*_*d*_) for patients with PD, at four time points (i.e., pre-, week 2, week 4, and post-training) during the balance-training program, in tasks with stance on rigid surface with eyes open (RO), and eyes closed (RC). Significant measures are in bold. Tukey *P* values are reported for post-hoc pairwise comparisons. The first time point, at which significant change appeared, and further time points, if that level of improvement retained, are marked with asterisk

**Fig. 2:**
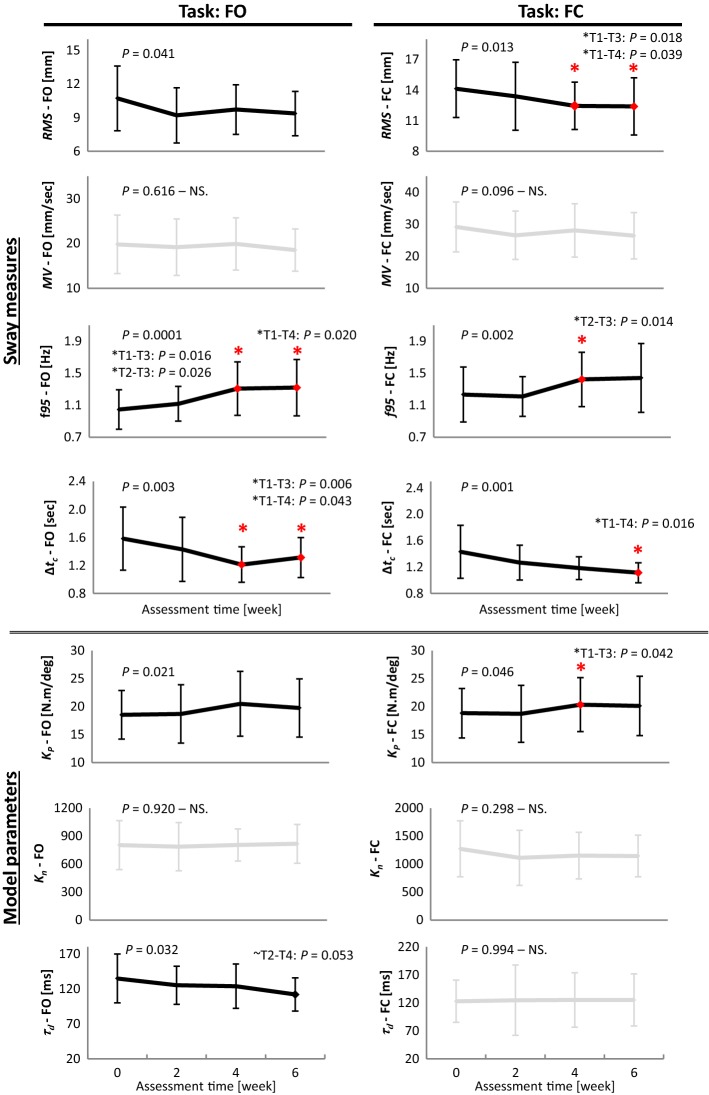
The pattern of improvements for sway measures (*RMS*, *MV*, *f95*, ∆*t*_*c*_) and model parameters (*K*_*P*_, *K*_*n*_, *τ*_*d*_) for patients with PD, at four time points (i.e., pre-, week 2, week 4, and post-training) during the balance-training program, in tasks with stance on foam with eyes open (FO) and eyes closed (FC). Significant measures are in bold. Tukey *P* values are reported for post-hoc pairwise comparisons. The first time point, at which significant change appeared, and further time points, if that level of improvement retained, are marked with asterisk

Furthermore, Figs. 1 and 2 show the pattern of improvements for the sway measures (*RMS*, *MV*, *f95*, ∆*t*_*c*_) and model parameters (*K*_*P*_, *K*_*n*_, *τ*_*d*_) in R-tasks and F-tasks, respectively. The first time point, at which each measure achieved significant change, and further time points, if maintained that level of change, are marked with asterisk. *K*_*D*_ and *K*_*I*_ did not significantly change in any tasks and were excluded from the figures (see Additional file 1, Fig. S1, for patterns of *K*_*D*_ and *K*_*I*_).

In R-tasks (Fig. 1, Table 2), only *MV* (RO: *P* = 0.010, *F* = 4.13; RC: *P* = 0.034, *F* = 3.09) and *K*_*n*_ (RO: *P* = 0.022, *F* = 3.48; RC: *P* = 0.035, *F* = 3.07) improved (increased significantly) after balance training (Fig. 1, bold plots). *MV* and *K*_*n*_ increased by 22.6% and 27.3%, in RO; and by 25% and 31.3% in RC, respectively. The improvement in flexibility-related measures, *K*_*n*_ and *MV*, was achieved late, at the end of the training program at week 6. In general, the statistical significance in *K*_*n*_ and *MV* was stronger in RO than in RC. As for measures related to stability, *f95*, *K*_*P*_*RMS*, and ∆*t*_*c*_ did not change after training in R-tasks. Patients also showed a trend toward gradual decline in time delay (*τ*_*d*_) in task RO (*P* = 0.059).

In F-tasks (Fig. 2, Table 3), *K*_*P*_ significantly increased (FO: 6.6%, *P* = 0.021, *F* = 3.50, FC: 6.9%, *P* = 0.046, *F* = 2.84), which reached significant changes from baseline, at week 4 (FC: *P* = 0.042). However, *K*_*P*_ ceased further improvements after week 4 and slightly returned to the baseline level. Likewise, *f95* significantly increased (FO: 25.7%, *P* = 0.0001, *F* = 8.11; FC: 17%, *P* = 0.002, *F* = 5.81), with similar early emergence of improvements at week 4 (FO: *P* = 0.016; FC: *P* = 0.014), which further remained at a steady level. Major improvements in *f95* achieved from week 2 to week 4 (Table 3). ∆*t*_*c*_, the other stability-related measure, showed decline after training in both F-tasks (FO: 17%, *P* = 0.003, *F* = 5.35; FC: 22.4%, *P* = 0.001, *F* = 6.59). ∆*t*_*c*_ in FO achieved improvements before the cessation of the training program (at week 4, *P* = 0.006) and did not further decrease; while in FC, it continued progression to the end of the balance-training program (at week 6, *P* = 0.016). Time delay, as in task RO, generally reduced in FO (*P* = 0.032, *F* = 3.15). In view of the developed balance performance as well as reduced *τ*_*d*_, abnormally large *RMS* in patients significantly decreased (FO: 12.6%, *P* = 0.041, *F* = 2.94; FC: 12.3%, *P* = 0.013, *F* = 3.90). *RMS* had an overall reduction in FO; yet in FC, *RMS* showed a significant early drop at week 4 (*P* = 0.018), which, similar to *f95*, did not further change and remained at that attained level. The flexibility-related measures, *K*_*n*_ and *MV*, in contrast to R-tasks, did not change in F-tasks.

None of the measures, neither in R-tasks nor in F-tasks, changed in the first 2 weeks of training (non-significant from pre to week 2). In fact, *MV* and *K*_*n*_ in R-tasks, and *K*_*P*_ and *f95* in F-tasks displayed a delay (the steady interval between pre and week 2) before rising to a new level (Figs. 1, 2). On the other hand, although changes in *RMS* and ∆*t*_*c*_ (in F-tasks, Fig. 2), as well as *τ*_*d*_ (in RO and FO, Figs. 1, 2), in the first 2 weeks, were statistically non-significant; they embarked on a quick change in their average values since the onset of the training program.

## Discussion

This study investigated the motor learning dynamics of the postural control in people with PD, using the unidimensional measures of stability and flexibility degree that we proposed in a previous study [37]. The pattern of improvements during a 6-week balance-training program in people with PD was assessed. The evaluated outcomes comprised clinical measures of functional balance and mobility, posturography measures, and parameters of a patient-specific postural control model (particularly, the stability—*K*_*P*_, and flexibility degree—*K*_*n*_). Findings demonstrated that the balance-training program resulted in continuous improvements in mobility- and flexibility-related measures such as TUG, 6MWT, Tinetti gait score; as well as *MV* and *K*_*n*_ (flexibility degree), which changed significantly in R-tasks. Furthermore, balance- and stability-related measures timed tandem stance with eyes open, step test, Tinetti balance score as clinical measures; *f95*, ∆*t*_*c*_, *RMS* on foam, *K*_*P*_ (stability degree) as posturographic and model-based measures showed an early improvement, in F-tasks, and reached a plateau before the end of the training program. The present study proposed a systematic approach to study the impact of specific training programs on postural disabilities in PD; and as such facilitates the design of new individualized and optimal interventions.

The observed improvement at mid-training, and from mid- to post-training for clinical measures of functional mobility implies a relatively constant improvement in mobility. Esculier et al. [24] also observed a continuous reduction in TUG for people with PD, at mid-training (week 3) and post-training (week 6) during an 18-session balance training. Improved TUG even after short-term interventions [38, 39] supports the possibility that TUG (i.e., mobility) in PD can improve rapidly. Furthermore, the abrupt and ongoing improvement of gait performance in people with PD was documented with excessively short gait trainings, besides long-term trainings with multi-assessment design. For instance, a minimum of 2-week gait training promoted walking speed and gait performance [15, 40]. In addition, continuing increase in walking capacity—6MWT, using multiple assessments during 24 weeks of treadmill training in PD, was observed [26]. This improvement, however, was not restricted to gait trainings; rather, short-term strength training [17] or resistance training [41] also caused increase in 6MWT in PD. At the same time, there exist studies, which found no improvement in mobility measures, even after long-term interventions due to the high initial values of the measures at the baseline or the unfocused, non-specific type of training that was applied [42, 43]. Considering the pivotal role that additional factors such as type and duration of interventions play, the above-mentioned conjecture cannot be generalized.

Our findings on clinical balance tests suggest an early improvement (at mid-training) in postural stability, with subsequent plateaued behavior for the rest of the balance-training program. Such behavior—Saturation pattern—was in part, consistent with the results of a few studies, which included a mid-training assessment during a training program [24, 25]. For instance, Esculier et al. [24] reported improvements at mid-training for Tinetti total score, which remained almost the same to the end of the balance training. Unfortunately, none of these articles clearly reported whether a statistically significant change occurred from mid- to post-training; hence, complicating the differentiation between Saturation and Continuous pattern in the second half of the program. In the same manner, Ganesan et al. [25] found improvements at mid- (session 8) and post-training (session 16) in Tinetti balance score. However, this improvement was 24.5% up to mid-training and merely 12% from mid- to post-training; suggesting a plateauing form in the second half of the training program (again not statistically tested). As a more objective test of balance, Stankovic [44] asserted that step test and tandem/one-leg stance more precisely discriminate the balance disorder in PD. We found no previous study, which investigated the mid-training changes in either step test or timed tandem stance. However, in a study by Nieuwboer et al. [45], Tandem-EO improved almost to its maximum score, following a minimum of 9 sessions (3 weeks) cueing training (as equal duration and sessions as our mid-training), which favors our results on early improvement of balance scores at mid-training. One may suspect that the Saturation pattern seen in these clinical scales might be the consequence of a natural ceiling effect. However, as for step test, a capability of up to 25 taps was recorded for healthy subjects [46], implying that saturation in step test at 17 taps for PD patients (Table 1) was caused by the limited learning capacity in PD and not the ceiling effect in the assessment measure. Although most balance tests exhibited early improvement followed by saturation, a few balance tests behave differently. FRT showed a Continuous pattern. It is plausible that clinical scales such as FRT are in fact assessing multiple tangled aspects of postural control, i.e., balance (or stability) and mobility (or in particular flexibility); considering the proven significant contribution of axial flexibility in FRT [16]. This may reiterate that the commonly used clinical tests have potential shortcomings such as being insensitive [4, 23], being multidimensional in measuring a mixture of contributors to postural control [8, 19], being confined by ceiling effects [47, 48], and being poorly defined in the level of the underlying constructs [8]. All these facts highlight the need to re-define current clinical measures.

Despite the equivocal results that may arise from clinical scales, the consistent set of postural sway measures along with the proposed model-based measures (stability and flexibility degree), provided clear conforming results. Findings revealed a constant improvement in flexibility-related measures, and early progress with plateaued behavior for stability-related measure. The increment in *MV* and *K*_*n*_ (flexibility degree) in R-tasks was characterized by a continuous improvement throughout sessions; nevertheless, it appeared significant almost late—only at week 6. Esculier et al. [24] also reported late improvement in *MV*, only at the end of the 6-week balance-training program. Interestingly, similar to our finding, *MV* in EC condition hardly improved as compared to EO condition [24]. Moreover, PD patients showed an accumulating capacity to improve the upper extremity movement velocity over a longer course of training (2-year progressive resistance training—PRE) [49]; suggesting the potential in flexibility and range-of-motion (ROM) features to improve continuously. Although both mobility- and flexibility-related measures exhibited a continuous progress, results indicated that flexibility, in contrast to mobility, reached significant changes at later times. Mobility advances sooner, likely because commuting to the rehabilitation center and participating in trainings, in turn, develop the physical and psychological well-being. In fact, the early improvements in mobility may be attributed to leaving the sedentary lifestyle; but its further improvements may be due to the gradual progress in other root factors such as flexibility. Nicely, Shen et al. [50] noticed that patients who dropped out a training program had lower mobility in comparison to non-dropout ones. While usual exercise guidelines (e.g., by American College of Sport Medicine—ACSM) emphasize on longer exercise duration to achieve sustained improvements in flexibility [4] (at least 6 weeks [15]), a minimum of 2 [40] to 4 weeks [23] intervention turned out to be sufficient to enhance mobility. It is noteworthy that flexibility-related measures were mainly reflected in R-tasks. Conversely, improved stability in the patients was mainly reflected in stability-related measures in F-tasks since these tasks challenge the stability more intensively.

The pattern of stability-related measures (*f95*, ∆*t*_*c*_, *K*_*P*_, *RMS*) in F-tasks was characterized by two main features: first, an early improvement during the first 4 weeks of training, and then a plateaued behavior in the remaining 2 weeks of the training. As for the early improvement of balance, one potential reason may be that fast strength gain occurs in muscles, during the first weeks of training, due to the neural adaptation and muscle fiber recruitment [17, 21, 47, 51]. Nonetheless, the neural adaptation appears as a transient response, during the first 2 weeks of training [21], which is shown to have transient central manifestation as well [11]. Apparently, after 2 weeks of training, the neural changes grow to physiological changes and muscular hypertrophy [52, 53]; which in turn translates to enough strength to significantly influence postural stability at week 4. It is well evidenced that enough muscular strength directly contributes to postural stability [9, 47, 54]. The developed stability over a short time span of 4 weeks is also in agreement with other studies which noticed improvements in balance performance (such as Berg balance scale, sensory organization test, limit of stability) by minimum of 4 weeks of training [23, 51, 55]. Furthermore, results revealed that the proposed model-based measures are more conservative than the postural sway measures, considering the smaller value of significance for *K*_*P*_ (or *K*_*n*_) as compared to *f95* and ∆*t*_*c*_ (or *MV*). This is because model-based measures are expressing some more subtle underlying neurophysiology of postural control.

The plateaued behavior in stability-related measures after some early rise was observed in some previous studies. Corcos et al. [49] noted such plateaued behavior in mean elbow flexion torque after 6 months, in favor of the PRE group compared to non-progressive control group which was even worsened over the 2-year training program. This is while both PRE and control group had shown similar strength gain during the first 6 months of training; indicating that strength gain is achievable to some extent, regardless of the training program. However, regarding the chronic feature of PD [8, 21], further strengthening demands more focused progressive programs. This observation supports the impression that the attainable strength and as such the learning capacity for postural stability in PD patients may be limited and have tendency to stop after a while. Likewise, Peterson et al. [28] claimed that people with PD may exhibit early, but not continued improvement in balance performance by training. In their study, the postural responses to translational perturbations in one-day practice were investigated in PD and healthy controls. Unlike healthy controls, improvements in people with PD occurred primarily in the first blocks of trials and then plateaued; whereas healthy controls gradually improved over all blocks of trials [28]. Other possible explanations for such behavior may be the insufficiency of the challenges and stimulus provided in the exercises, or the induced fatigue and detraining effects during the two closing weeks of the program [14, 47, 56]. However, it is less probable in our study since we employed a progressive difficulty level for the exercises throughout sessions. Interestingly, unlike *RMS* and *f95*, which plateaued at a steady level, *K*_*P*_ and ∆*t*_*c*-_-FO relatively reverted back to baseline. There are also studies that addressed such regress-to-baseline pattern in postural sway measures during a training program [56, 57]. However, these results should be interpreted cautiously, given the *inherent bounds*, or the maximum/minimum *normal value* that any measure such as *K*_*P*_, *f95*, etc. can attain and may stagnate at that level.

As an intriguing finding, our results revealed that improvements in some measures (e.g. *MV*, *K*_*n*_, ∆*t*_*c*_, *f95*) occurred sooner (or with stronger significant difference) in EO condition than the EC condition, likely because EC tasks are more difficult. From this perspective, the continuous improvement in Tandem-EC and ∆*t*_*c*_-FC, compared to the saturated improvement in Tandem-EO and ∆*t*_*c*_-FO, is explained. Similarly, *τ*_*d*_ showed decline only in EO tasks (RO and FO).

Such observations might suggest that an optimal training program for postural control in PD should focus on stability during the first weeks of training, and enjoying higher intensity of mobility and flexibility exercises during the ending weeks of training. However, asserting an established optimal training regimen still needs more comprehensive and well-documented information on the learning dynamics of postural control during other different training programs (e.g., strength training, gait training, resistance training, etc.), using the proposed approach.

This study had limitations. Some of the patients in the study were taking psychotropic drugs (i.e., antidepressants and benzodiazepines) that may induce impairments in balance and postural control. Furthermore, the inclusion of a PD control group as well as a healthy control group as to limit the placebo effects is lacking. In addition, it is intriguing for future studies to design longer interventions with more assessment times during the intervention, as well as during the follow-up inspection. As such, future studies can discover an analytical formula for learning dynamics and dose–response relationships of postural control. Using longer training programs may also reveal the change patterns for other measures such as *K*_*I*_ and *K*_*D*_, which was non-significant in the current study. Future studies also can employ targeted exercises to define the exact added value of each modality.

## Conclusions

The balance-training program resulted in early improvement of postural stability with plateaued behavior, in PD. On the other hand, flexibility-related measures took longer time to show improvement, yet exhibited a continuous progression during the training. Furthermore, improvements in mobility were achieved early at mid-training, and continued to improve to the end of the training. Taken together, the proposed framework provides a basis for the systematic analyses of motor learning dynamics of postural control in PD, which facilitates the future design of optimal training programs. Furthermore, the framework benefits from quantitative measures and a patient-specific model, which prepare the ground for design of individualized training programs.

## Methods

### Participants and balance-training Program

Twenty patients with PD, diagnosed as outlined by the UK Parkinson’s Disease Society Brain Bank Criteria [58] (Hoehn and Yahr ≤ 3, Mini-Mental State Examination score ≥ 24), who had no other comorbidities (e.g., neurological, musculoskeletal disorders, etc.) were included in the study (Table 4). Patients were eligible if they were able to walk independently for 10 m, and were on stable dopaminergic therapy. All patients provided written informed consent according to the Declaration of Helsinki. The study was approved by the local ethics committee.

**Table 4:** Patients’ characteristics

Characteristic	PD patients (*n* = 20)
	Mean ± standard deviation
Age (years)	63.3 ± 7.5
Gender (male:female)	15: 5
Height (m)	1.67 ± 0.08
Weight (kg)	69.7 ± 14.7
Disease duration (years)	8.15 ± 4.8
Most affected side (right:left)	14: 6
Disease severity (Hoehn and Yahr)	1.8 ± 0.7
Medications	
Madopar/Levodopa, No. (%)	20 (100)
Dopamine agonists, No. (%)	6 (30)
Antidepressants, No (%)	4 (20)
Benzodiazepines, No (%)	3 (15)

The patients received 18 sessions of balance exercises (3 days/week for 6 weeks) in an outpatient rehabilitation center. Each session lasted for 60–90 min, with 10-min warm-up followed by 20 min of conventional rehabilitation (such as stretching, range-of-motion exercise, body-weight strengthening of hip and ankle, volitional/large stepping, forward/backward/sideways walking), and 30–60 min of balance exercises. The balance exercises included both overground balance exercises and device-based exercises. A laboratory-developed device, *Balance Robot*, was used for the device-based exercises. The *Balance Robot* consisted of a motorized support surface, which applied controlled tilt motion in all directions, and equipped with a customized force plate to provide visual feedback of the COP on a monitor in front of the patient. The exercises with the *Balance Robot* included Limit of Stability (LOS), Random Control, and Postural Stability [59, 60]. In the LOS exercise, the patients had to lean to different directions, in order to hit 8 targets using their COP. The targets were located on a circle around, and were displayed on the monitor. The patients had to reach and hit the blinking target which was randomly selected form the eight. The patients had to lean back and to re-position their COP at the center after successfully hitting each target (a maximum of 60 s was considered for each trial; however, the patients were asked to hit the targets as quickly as possible). The size of the targets was gradually shrunk, and the distance of the targets was progressively increased, from session to session. In Random Control exercise, a moving circle was shown to the patients on the monitor, and they were asked to follow the circle, and to keep their COP within the circle. The circle moved randomly in all directions on the screen and within each patient’s affordable space (up to 80% of the patient’s maximum lean in different directions, which was calibrated at the beginning of each session). The circle was shrunk in size and speeded up in moving throughout sessions, as to increase the difficulty level of the exercises. Postural Stability exercises included maintaining balance (i.e., keeping the COP as close as possible to the center position) while standing on the disturbing support surface with two levels of disturbance (Dist1, Dist2). Disturbances included sequences of tilt motions with random-amplitude and random-speed in the anterior–posterior direction. The amplitude was randomly set in the range of 1° to 7° in Dist1, and 2° to 11° in Dist2. The speed was also randomly selected from the range of 1 deg/s to 10 deg/s in Dist1 and to 15 deg/s in Dist2. The two exercises with *Balance Robot* (LOS, and Random Control) were performed on ‘No disturbance’ during weeks 1–2, on Dist1 during weeks 3–4, and on Dist2 during weeks 5–6 (see Appendix and Additional file 1, for the detailed exercises of each session and the training progression). In addition, patients were asked to maintain balance in response to unexpected toe-down/up 7° tilt perturbations in all sessions. The overground balance exercises involved maintaining balance in different stance conditions (quiet stance, semi-tandem stance, tandem stance, one-leg stance), while receiving sensory stimulations (on foam, with closed eyes, with movements of the head), or while performing upper extremity tasks (throwing ball, reaching, etc.). Training progression throughout sessions was provided by reducing or manipulating sensory information, necessary to obtain balance (see Appendix and Additional file 1, for detailed overground balance exercises). The exercises were designed based on the task difficulty, which progressed through sessions to remain challenging, while considering the patients’ safety. The patients wore a safety harness while standing on the *Balance Robot*, also with safety handles around, and performed exercises under direct supervision of a therapist. Patients were allowed to rest between exercises, as needed. All patients completed the balance-training program and none of them reported any side effects.

### Testing procedure and outcome measures

*Multipoint*-*assessment design* The clinical assessments were performed three times, at baseline (pre-training), mid-training (i.e., week 3), and after the completion of the training program (post-training, week 6). In addition, experimental assessments were performed, using static posturography, at four time points: pre-training, post-training (week 6), and two time points during the training program (at weeks 2 and 4). All assessments and training sessions were held while patients were ON-medicated, i.e., about 1–2 h(s) after taking their usual dopaminergic medication.

*Clinical assessment* [20, 48] Clinical assessments consisted of functional reach test (FRT), Tinetti performance-oriented assessment tool (balance section), timed tandem stance with eyes open (Tandem stance—EO) and closed (Tandem stance—EC), and step test [44] to examine functional balance; as well as Timed Up and Go test (TUG), 6-min walk test (6MWT), and Tinetti performance-oriented assessment tool (gait section), for the assessment of functional mobility. Tandem stance was performed with the right and then left leg, in the front position and until patients reached a maximum of 60 s in each test; and then the time of both legs was summed as one scale (with maximum score of 120 s).

*Experimental assessment* The whole experimental assessment procedure is completely similar to the method of our previous study and described in detail in [37]. For static posturography, the center-of-pressure (COP) of patients was recorded for 70 s at 1 kHz, while standing on a force plate (type 9260AA6, Kistler Instrument AG, Winterthur, Switzerland) in eight trials: quiet stance on rigid surface with eyes open and closed (RO, RC); and on 10.5-cm-thick foam with eyes open and closed (FO, FC); each in two repetitions. The order of tasks was randomized for each patient to avoid any bias caused by learning effects. Patients were allowed to have sufficient rest intervals between the trials, if they needed.

Four postural sway measures were calculated from the COP data for each patient and each task (5–65 s of each trail and averaged for each task): root mean square (*RMS*) of the COP displacement, mean velocity (*MV*), the frequency associated with the 95% of the total power (*f95*), and the time coordinate of the critical point in the stabilogram diffusion function (SDF) diagram (∆*t*_*c*_) [61]. *RMS* provides a measure of sway amplitude, which is normally larger in PD patients [32]. *MV* also reflects the degree to which patients regulate the spontaneous sway in a flexible manner [37, 62]. Higher *MV* reflects higher flexibility. *f95* and ∆*t*_*c*_, as frequency-domain measures, are associated with the ankle stiffness. Greater *f95* (smaller ∆*t*_*c*_) indicates higher stiffness. However, these measures are the overall outcome of the interconnected underlying neurophysiological mechanisms, and therefore were projected onto a postural control model to separate stability and flexibility degree [37].

**Fig. 3:**
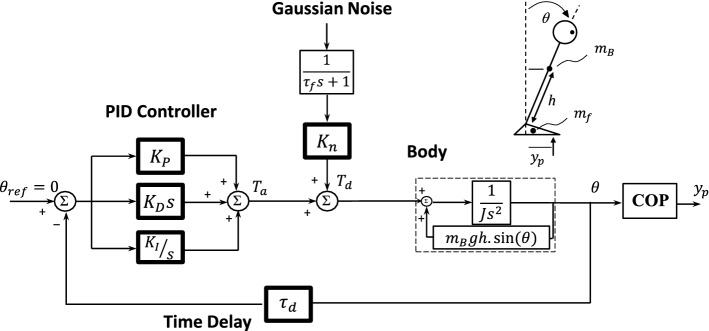
The patient-specific postural control model of PD. The model consisted of human ‘Body’, CNS in form of a PID controller, and time delay (*τ*_*d*_). The ‘Body’ was modeled by an inverted pendulum with all mass (*m*_*B*_) centered at the height of *h* (which were adjusted patient-specifically). *J*, moment of inertia of body around ankle axis. The COP displacement (*y*_*p*_) was calculated from the body sway angle (*θ*) considering the feet mass (*m*_*f*_ = 2.01 kg), which is fully described in [37]. The CNS was modeled by a PID controller: *K*_*P*_ (proportional gain—quantifies the stability degree), *K*_*D*_ (derivative gain), *K*_*I*_ (integral gain). *T*_*a*_, corrective ankle torque; *T*_*d*_, disturbance torque; *K*_*n*_, internal disturbance torque gain which quantifies the flexibility degree; *τ*_*f*_ = 100 s, time constant for low-pass filter

Based on the COP-based sway measures, the parameters of a patient-specific postural control model of PD (Fig. 3) were estimated through an optimization algorithm (i.e., *K*_*P*_, *K*_*D*_, *K*_*I*_, *K*_*n*_, *τ*_*d*_) [37]. The model consists of an inverted pendulum, which is defined by body mass *m*_*B*_ at height *h*; a PID controller (*K*_*P*_, *K*_*D*_, *K*_*I*_) representing the central nervous system (CNS) control performance; and a time delay *τ*_*d*_, which corresponds to the time delay that CNS takes to respond. A disturbance torque (*T*_*d*_) in form of a Gaussian noise (filtered by a low-pass filter with time constant *τ*_*f*_ = 100 s) is injected into the control loop to mimic the spontaneous sway—scaled by gain *K*_*n*_. The output of the model is the COP displacement *y*_*p*_, calculated from the body sway angle (*θ*) [37].

From the model point of view, *K*_*P*_ and *K*_*n*_ exclusively quantify the stability and flexibility degree, respectively, that contribute to the spontaneous sway. *K*_*D*_ expresses the ankle damping, and *K*_*I*_ denotes the amount of effort from the CNS to correct for undesired steady deviation from the upright position. Three parameters *K*_*P*_, *K*_*D*_, and *K*_*I*_ adjust the amount of the corrective ankle torque (*T*_*a*_). Accordingly, *K*_*P*_ is an estimate of the ankle stiffness and therefore greater *K*_*P*_ is associated with larger *f95* (smaller ∆*t*_*c*_). On the other hand, *K*_*n*_ exclusively adjusts sway amplitude, affecting *MV* and *RMS* independent from changes in control parameters (i.e., *K*_*n*_ exclusively quantifies the ‘flexibility’ degree, regardless of changes in ‘stability’). Greater *K*_*n*_—more flexibility—manifests in larger *MV*, the phenomenon which is observed in PD after rehabilitation, due to the amelioration of rigidity (improvement in flexibility) [24, 37, 63]. Postural sway measures reflect an overall performance of the postural control. As an instance, *RMS* is simultaneously adjusted by *K*_*P*_ (stability degree), *K*_*n*_ (flexibility degree), and *τ*_*d*_. Therefore, we used these model-based measures to prevent misinterpretation of simple postural sway measures like *RMS* [37, 64]. The model-based measures are sensitive enough to detect improvements after a balance-training program [36, 37].

The sway measures and model parameters constituted the two sets of stability-related (*f95*, ∆*t*_*c*_, *K*_*P*_—stability degree) and flexibility-related measures (*MV*, *K*_*n*_—flexibility degree). Improvement in flexibility-related measures (*MV* and *K*_*n*_) is significant on tasks with stance on rigid surface (R-tasks); conversely, improvements in measures related to stability (*f95*, ∆*t*_*c*_, and *K*_*P*_) are significant in foam standing tasks (F-tasks) [17, 37, 65].

All sway measures and model parameters were calculated for each patient in each task, and at each time point of experimental assessment (i.e., pre-, week 2, week 4, and post-training).

### Statistical analysis

TUG, which has shown a high validity and reliability in PD [66], was chosen for the sample size calculation. A sample size of 18 was required for the study to have 80% statistical power, and 95% confidence level (*P* < 0.05), considering the TUG results of a pilot study. By correcting for a potential loss of 10% as to drop out from the program, we included 20 patients in the study. The further power calculation of the current results (found to be 95% at the end of study) indicated the sufficiency of the sample size. The normal distribution of all clinical and experimental measures was tested using the Shapiro–Wilk normality test. All sway measures and model parameters were randomly distributed. Among clinical measures, Tinetti balance score, Tinetti gait score, and Tandem stance–EO were non-normal, which were log-transformed before being used in the statistical analysis. The temporal improvements for each of the clinical and experimental outcomes were studied individually in each task. For this purpose, repeated measure analysis of variance (ANOVA) with one factor (*Time*) was performed for each of the clinical and postural sway measures, as well as the model parameters in each task. Factor *Time* includes three levels for the clinical measures (pre, mid, post); and four levels for the sway measures and model parameters (pre, week 2, week 4, post). The Tukey test was used for post-hoc multiple pairwise comparisons between time points. Statistical significance was set at *P* < 0.05.

## Supplementary information

**Additional file 1: Figure S1.** The pattern of improvements for (A) *K*_*D*_ and (B) *K*_*I*_, for patients with PD, at four time points (i.e. pre-, week 2, week 4, and post-training) during the balance-training program, and in all four tasks of stance on rigid surface (RO, RC), and stance on foam (FO, FC). All the changes were non-significant. **Table S1.** Details of the balance-training program.

## Data Availability

The data used during the current study are available from the corresponding author on reasonable request.

## Appendix

See Table 5.

**Table 5:** The balance-training program

Week	Dist. level*	Exercises with *Balance Robot*			Overground balance exercises and conventional exercises
		Limit of stability (LOS)^a^ target (size, distance)^b^	Random control^c^ circle (size, speed)^d^	Postural stability^e^	
1–2	No Dist.	Size: 1–5 distance: 1–3	Size: 1–5 Speed: 1–4	Week 1: – Week 2: Dist1	Stance on rigid surface with combination of these conditions (from easier to more difficult): Eyes open/eyes closed Semi-tandem/tandem/one-leg With ball in hands Walking: In tandem gait with eyes open and closed, Forward, backward, sideways, Step around and over obstacles Combined with sit-to-stand-up
3–4	Dist1	Size: 1–5 Distance: 1–3	Size: 1–5 Speed: 1–4	Week 3: Dist 1 week 4: Dist 2	
					Standing on foam with combination of these conditions (from easier to more difficult): Eyes open/eyes closed Tandem/one-leg/one-leg and rolling a rod, turning a rod, or writing their name, or rhythmically tap a step of 7.5 cm height with other leg With ball in hands, throwing ball to different directions, given shoulder pulls by the trainer, rotating head and the trunk, squat Combined with sit-to-stand-up Walking: Combined with sit-to-stand-up Crossing obstacles, kicking a ball, with ball in hands, throwing ball to different directions, rotating head and the trunk
5–6	Dist 2	Size: 1–5 Distance: 1–3	Size: 1–5 Speed: 1–4	Week 5: Dist 2 week 6: -	

^a^LOS exercise repetitions in each session: 3–7 rep

^b^Target sizes: 1–5 (size 1 is the largest target circle, and size 5 the smallest target circle); Target distances: 1–3 (distance 1 is the nearest distance at 50% of each patient’s maximum forward lean; distance 2 is at 80% of each patient’s maximum forward lean; and distance 3 is at 100% of each patient’s maximum forward lean. Distances were pre-calibrated and set according to each patient’s maximum forward lean at the beginning of each session

^c^Random Control exercise repetitions in each session: 2–3 rep

^d^Circle sizes: 1–5 (size 1 is the largest circle size, and size 5 the smallest circle size); Circle speed: 1–4 (speed 1 is the slowest, and speed 4 is the fastest almost affordable speed)

^e^Postural Stability exercise repetitions in each session: 2–3 rep. The Postural Stability exercise was performed on the random tilt disturbances of support surface in anterior–posterior direction, either with setting Dist1 or Dist2 as described below

* Two exercises with *Balance Robot*, i.e., Limit of Stability (LOS) and Random Control were performed on an stationary support surface (‘No Disturbance’) during weeks 1–2, or on the disturbing support surface with two levels of ‘Dist1’, and ‘Dist2’, during weeks 3–6. The disturbances were in the form of random-amplitude and random-speed sequences of tilt motions in the anterior–posterior direction. The amplitude was randomly set in the range of 1° to 7° in Dist1, and 2° to 11° in Dist2. The speed was also randomly selected from the range of 1 deg/s to 10 deg/s in Dist1 and to 15 deg/s in Dist2

## Abbreviations

PD: Parkinson’s disease
COP: Center-of-pressure
RMS: Root mean square
MV: Mean velocity
EO: Eyes open
EC: Eyes closed
RO: Rigid surface with eyes open task
RC: Rigid surface with eyes closed task
FO: Foam surface with eyes open task
FC: Foam surface with eyes closed task
R-tasks: Rigid-surface tasks
F-tasks: Foam-surface tasks
TUG: Timed Up and Go test
FRT: Functional reach test
6MWT: Six-minute walk test
PRE: Progressive resistance exercise
LOS: Limit of stability
