# Supplementary material for: Postural control learning dynamics in Parkinson’s disease: early improvement with plateau in stability, and continuous progression in flexibility and mobility
Source: Biomed Eng Online. 2020 May 11;19:29. doi: 10.1186/s12938-020-00776-1 (PMC7216342; doi:10.1186/s12938-020-00776-1)
Supplement: Supplementary file 1 — Additional file 1: Figure S1. The pattern of improvements for (A) KD and (B) KI, for patients with PD, at four time points (i.e. pre-, week 2, week 4, and post-training) during the balance-training program, and in all four tasks of stance on rigid surface (RO, RC), and stance on foam (FO, FC). All the changes were non-significant. Table S1. Details of the balance-training program. [file 12938_2020_776_MOESM1_ESM.docx]

***Supplementary Material***

**­­­­ Postural control learning dynamics in Parkinson’s disease: early improvement with plateau in stability, and continuous progression in flexibility and mobility**

**Zahra Rahmati, Saeed Behzadipour*, Alfred C. Schouten, Ghorban Taghizadeh, Keikhosrow Firoozbakhsh**

*** Correspondence:** Corresponding Author: [behzadipour@sharif.edu](mailto:behzadipour@sharif.edu)

1. **The pattern of improvements in *K_D_* and *K_I_***

Figure S1 - The pattern of improvements for (A) *K_D_* and (B) *K_I_*, for patients with PD, at four time points (i.e. pre-, week 2, week 4, and post-training) during the balance-training program, and in all four tasks of stance on rigid surface (RO, RC), and stance on foam (FO, FC). All the changes were non-significant.

1. **Details of the balance-training program**

| Dist. level* | Session No. | Exercises with *Balance Robot* | | | Overground balance exercises  and Conventional exercises^f^ |
| --- | --- | --- | --- | --- | --- |
|  |  | Limit of Stability (LOS)^a^  target (size, distance)^b^ | Random Control^c^  circle (size, speed)^d^ | Postural Stability^e^ |  |
| No Dist. | 1 | size: 1  distance: 1 | size: 1  speed: 1 | - | - walking in tandem gait with a ball in hands,  - walking backward with a ball in hands |
|  | 2 | size: 3  distance: 1 | size: 3  speed: 2 | - | - step around obstacles  - step around obstacles while kicking a soccer ball |
|  | 3 | size: 5  distance: 2 | size: 4  speed: 3 | Dist1 | - sit-to-stand-up, crossing an obstacle, and sit back  - sit-to-stand, crossing an obstacle, and sit back with kicking a soccer ball, |
|  | 4 | size: 4  distance: 3 | size: 4  speed: 4 | Dist1 | - one-leg stance with eyes open,  - tandem stance with closed eyes,  - sideway walking |
|  | 5 | size: 5  distance: 3 | size: 5  speed: 4 | Dist1 | - walking in tandem gait with closed eyes,  - walk backward in tandem stance with eyes open,  - one-leg stance with eyes closed, |
| Dist1 | 6 | size: 1  distance: 1 | size: 1  speed: 1 | Dist1 | - walking in tandem gait with closed eyes and with a ball in hands,  - crossing obstacles with walking sideway, |
|  | 7 | size: 3  distance: 2 | size: 2  speed: 2 | - | - sit-to-stand-up, walking forward to pick blue pens from the ground, turn back, place the pens, and sit down,  - Tandem stance on foam with eyes open, |
|  | 8 | size: 5  distance: 2 | size: 3  speed: 3 | Dist1 | - one-leg stance with closed eyes,  - tandem stance and reach to different directions, |
|  | 9 | size: 1  distance: 3 | size: 4  speed: 3 | Dist2 | - stance on one leg and roll a rod with the other leg,  - rhythmically raise thigh to hit the hand palm,  - given shoulder pulls by the trainer, while standing with eyes open and a ball in hands, |
|  | 10 | size: 3  distance: 3 | size: 4  speed: 4 | Dist2 | - tandem stance on foam with eyes closed  - sit-to-stand-up on one leg |
|  | 11 | size: 5  distance: 3 | size: 5  speed: 4 | Dist2 | - sit-to-stand-up and step over large obstacles, turn, step over large obstacles, and sit back,  - sit-to-stand-up and stand on one leg with eyes closed |
| Dist2 | 12 | size: 5  distance: 1 | size: 1  speed: 1 | Dist2 | - sit-to-stand-up on foam,  - abduction/adduction of hip while standing  - sit-to-stand-up with one leg with eyes closed |
|  | 13 | size: 2  distance: 2 | size: 3  speed: 1 | Dist2 | - sit-to-stand-up with different speeds,  - stance on one leg and rotate a rod with other leg,  - given shoulder pulls by the trainer, while standing on a foam with eyes open, |
|  | 14 | size: 5  distance: 2 | size: 2  speed: 2 | Dist2 | - sit-to-stand-up on a foam with eyes open  - stance on one leg and write his/her name with the other leg, |
|  | 15 | size: 1  distance: 3 | size: 2  speed: 3 | - | - tandem stance and throwing ball to different directions,  - given shoulder pulls by the trainer, while standing on a foam with eyes closed,  - sit-to-stand-up on a foam with eyes closed |
|  | 16 | size: 2  distance: 3 | size: 3  speed: 3 | - | - standing on a foam and squat,  - stance on one-leg and place the other foot on a 15 cm-height step, with a ball in hands, and repeat it. |
|  | 17 | size: 4  distance: 3 | size: 4  speed: 4 | - | - standing on a foam and tap a 7.5 cm-height step in the front with one leg.  - sit-to-stand-up and cross three obstacles with a ball in hands, and turning back,  - walking with a ball in hands, and rotating hands to the sides, |
|  | 18 | size: 5  distance: 3 | size: 5  speed: 4 | Dist2 | - Standing on an foam with a ball in hands, while rotating hands to the sides and back,  - stance on a foam and throwing ball to different directions, |

^a^ LOS exercise repetitions in each session: 3-7 rep.

^b^ Target sizes: 1-5 (size 1 is the largest target circle, and size 5 the smallest target circle); Target distances: 1-3 (distance 1 is the nearest distance at 50% of each patient’s maximum forward lean; distance 2 is at 80% of each patient’s maximum forward lean; and distance 3 is at 100% of each patient’s maximum forward lean. Distances were pre-calibrated and set according to each patient’s maximum forward lean at the beginning of each session.

^c^ Random control exercise repetitions in each session: 2-3 rep.

^d^ Circle sizes: 1-5 (size 1 is the largest circle size, and size 5 the smallest circle size); Circle speed: 1-4 (speed 1 is the slowest, and speed 4 is the fastest almost affordable speed).

^e^ Postural Stability exercise repetitions in each session: 2-3 rep. The Postural Stability exercise was performed on the random tilt disturbances of support surface in anterior-posterior direction, either with setting Dist1 or Dist2 as described below.

^f^ Conventional rehabilitation also included trunk, hand, head and different types of upper extremity exercises

* Two exercises with *Balance Robot*, i.e. Limit of Stability (LOS) and Random Control were performed on an stationary support surface (‘No Disturbance’) during sessions 1-6, or on the disturbing support surface with two levels of ‘Dist1’, and ‘Dist2’, during sessions 11-18. The disturbances were in the form of random-amplitude and random-speed sequences of tilt motions in the anterior-posterior direction. The amplitude was randomly set in the range of 1° to 7° in Dist1, and 2° to 11° in Dist2. The speed was also randomly selected from the range of 1 deg/sec to 10 deg/sec in Dist1 and to 15 deg/sec in Dist2.
